# Intake of Meals Containing High Levels of Carbohydrates or High Levels of Unsaturated Fatty Acids Induces Postprandial Dysmetabolism in Young Overweight/Obese Men

**DOI:** 10.1155/2015/147196

**Published:** 2015-11-02

**Authors:** Edyta Adamska, Lucyna Ostrowska, Joanna Gościk, Magdalena Waszczeniuk, Adam Krętowski, Maria Górska

**Affiliations:** ^1^Clinical Research Centre, Medical University of Bialystok, M.C. Skłodowskiej-Curie 24A, 15-276 Bialystok, Poland; ^2^Department of Dietetics and Clinical Nutrition, Medical University of Bialystok, Mieszka I-go 4B, 15-054 Bialystok, Poland; ^3^Centre for Experimental Medicine, Medical University of Bialystok, M.C. Skłodowskiej-Curie 24A, 15-276 Bialystok, Poland; ^4^Department of Endocrinology, Diabetology and Internal Medicine, Medical University of Bialystok, M.C. Skłodowskiej-Curie 24A, 15-276 Bialystok, Poland

## Abstract

Postprandial metabolic response depends on the meals' components and can be different in normal weight and obese people. However, there are some discrepancies between various reports. The aim of this study was to determine the metabolic response after intake of standardised meals with various fat and carbohydrate contents and to determine the differences among normal weight and overweight/obese individuals. The study group comprised 46 healthy men. The participants were divided into two groups and study was carried out using a crossover method. Group I received high- and normal-carbohydrate meals, whereas group II received high-carbohydrate and high-fat meals. Glucose, insulin, triglyceride, and free fatty acids levels were measured at fasting state and at 30, 60, 120, 180, and 240 minutes after meal intake. Despite the lack of differences in glucose levels, insulin levels were higher among overweight/obese individuals after each meal. TG and FFA levels were higher after normal-carbohydrate and high-fat meals. Moreover, in overweight/obese young men after high-fat meal intake postprandial hypertriglyceridemia was observed, even if meals contained predominantly unsaturated fatty acids, and fasting triglycerides levels were in normal range. The conducted study showed that postprandial metabolic response depends not only on the meal macronutrient content but also on the current body mass index (BMI).

## 1. Introduction

Obesity is a chronic metabolic disease and a growing medical issue with a global reach. In the last 30 years, a dramatic increase in number of people suffering from obesity has been observed. The prognosis for the future is alarming, as it is believed that, with current trends, over a billion of people will have been obese by the year 2030, and another 2 billion will have been overweight [1]. The main causes of obesity are positive energy balance and unbalanced diet. Recently, the trends of diets have become more and more encouraged and understanding the health benefits resulting from complying with appropriately composed diet may help to reduce the progress of obesity and its consequences development. Considering that meals are usually consumed at least three times a day, where assimilation of nutrients usually takes around 5-6 hours, the human body remains in the postprandial state for the greater part of a day [2]. Meal ingestion causes a series of metabolic reactions, often referred to as “postprandial dysmetabolism,” which is related to coronary artery diseases and cardiovascular incidents [3–5]. To reduce the risk of cardiovascular disease it is recommended to replace the saturated fatty acids (SFA) with unsaturated fats, and this action seems to be more important than reduction in total fat intake [6–8]. It has been demonstrated that high-monounsaturated fatty acids (MUFA) diet improves postprandial metabolic response [9] and MUFA, as a replacement for SFA, provides a greater reduction in risk of coronary artery disease than carbohydrates [10]. Lozano et al. showed that people with BMI greater than 26.18 kg/m^2^ benefit from the consumption of MUFA coming from oil of olives, which came down to lowering their levels of TG-rich lipoproteins at the postprandial state [11]. On the other hand, the test meal studies confirm that high-fat meals have an adverse effect on postprandial vascular function; however the evidence for effects of high-fat meals rich in unsaturated fats is limited and inconclusive [12]. Moreover, it is worth mentioning that the postprandial metabolic changes, which depend on the composition of a meal, can be also different in overweight/obese people [11, 13, 14]. Ingestion of meals, which may have clinical and metabolic benefits, is crucial in prophylaxis and treating of obesity and its clinical consequences.

The aim of this study was to evaluate the metabolic changes after intake of meals with various fat and carbohydrate contents, using a crossover method, and to analyse the differences between postprandial metabolic responses among normal weight and overweight/obese healthy men. It was expected to discover early postprandial metabolic disturbances in young overweight/obese men in postprandial state, even if the baseline results were in normal range.

## 2. Experimental Methods

### 2.1. Study Participants

46 men participated in the study: 23 with normal weight (NW) and 23 were overweight/obese (OO) in the age range of 21–58 years. The study was conducted according to the guidelines laid down in the Declaration of Helsinki and all procedures involving patients were approved by the local Ethics Committee of the Medical University of Bialystok (Poland) and a written informed consent was obtained from all participants. The participants of the study did not suffer from any glucose metabolism disorders, endocrine disorders, renal or liver failure, and digestive system diseases, did not undergo any gastroenterological and bariatric surgeries or procedures, and did not suffer from any other diseases which could influence the results. People who had received pharmacological treatment or had used any other products with undocumented or unknown influence on metabolism were excluded from the study. Taking into consideration the fact that the levels of some factors can be characterised by sexual dimorphism and its analysis was not the aim of the study, only male participants were selected for the study group, in order to obtain reliable results.

**Figure 1:**
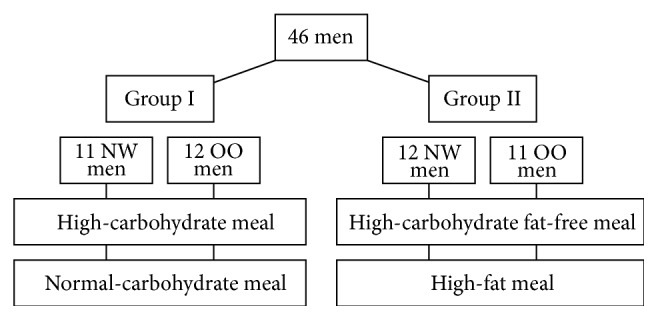
Study design. NW: normal weight; OO: overweight/obese.

Based on BMI, the participants were divided into two groups: men with normal weight (NW, BMI < 25.0 kg/m^2^) and men that were overweight/obese (OO, BMI ≥ 25.0 kg/m^2^). Subsequently, the participants were randomly divided (randomisation using sampling without replacement) into two experimental groups (the study design is presented in Figure 1 and the characteristic of the groups is presented in Table 1). The study was carried out using the crossover method.

Group I (11 NW men and 12 OO men) received a high-carbohydrate (HC) meal (450 kcal), 89% of energy coming from carbohydrates, 11% coming from protein, and 0% coming from fat (Nutridrink Fat Free, Nutricia, Poland), and, then, after a period of 1-2 weeks received a meal with normal-carbohydrate (NC) content (450 kcal), 45% of energy coming from carbohydrates, 30% coming from protein, and 25% coming from fat: 12,4% SFA, 59,0% monounsaturated fatty acids (MUFA), 28.7% polyunsaturated fatty acids (PUFA), and n-6/n3 ratio 5,11 (Cubitan, Nutricia, Poland).

Group II (12 NW men and 11 OO men) received a HC (fat-free) meal (450 kcal), 89% of its energy coming from carbohydrates, 11% coming from protein, and 0% coming from fat (Nutridrink Fat Free, Nutricia, Poland), and, then, after a period of 1-2 weeks received a high-fat (HF) meal (450 kcal), 4% of energy coming from carbohydrates, 0% coming from protein, and 96% coming from fat: 10.7% SFA, 60.7% monounsaturated fatty acids (MUFA), 28.6% polyunsaturated fatty acids (PUFA), and n-6/n3 ratio 5,02 (Calogen, Nutricia, Poland).

The participants were asked not to change their diet and daily physical activities during the study.

### 2.2. Study Procedure

The participants were arriving at the lab at 08:00–08:30 in the morning, in the fasting state, with at least 12 hours since their last meal. The following procedures were carried out: height and weight measurements and body fat content measurement (using the bioimpedance method, InBody 220 Biospace, Korea). A peripheral venous catheter was placed in the elbow crook and venous blood was drawn in order to determine fasting blood glucose and insulin and free fatty acids (FFAs) and triglyceride (TG) levels. The participants then received a randomly selected meal (at room temperature) and were advised to consume it within 10 minutes. 30, 60, 120, 180, and 240 minutes after meal intake the venous blood was drawn in order to measure the above-mentioned factors levels again.

### 2.3. Laboratory Tests

The specimen was drawn and prepared for testing in accordance with the recommendations provided by the laboratory kit producers. The assay of particular levels was carried out immediately after drawing the specimen, while the remaining ones were determined after the specimen was obtained for the whole period of tests. The specimen was stored in accordance with the recommendations of the producers until it was tested, at temperature of −20°C/−80°C. The particular factors levels were determined using the following methods: glucose-hexokinase enzymatic colorimetric assay (Cobas c111, Roche Diagnostics Ltd., Switzerland), insulin-immunoradiometric assay (Insulin, IRMA, DiaSource, Belgium; Wallac Wizard 1470 Automatic Gamma Counter, PerkinElmer, Life Science, Turku, Finland), TG-enzymatic colorimetric assay (Cobas c111, Roche Diagnostics Ltd., Switzerland), FFAs-enzymatic assay (FFA ELISA, Zen-Bio, Czech Republic; *μ*Quant, BioTek Instruments Inc., Winooski, Vermont, USA).

### 2.4. Statistical Analysis

In order to obtain the general characteristics of the data, arithmetic mean and standard error of the mean were calculated for all numerical features, which were treated as dependent variables in subsequent steps of the analysis. The aim of the study was to check whether there is a statistically significant postprandial metabolic response depending on meals' components. There were two main null hypotheses stated: (1) different types of meals (differentiated by main component of the meal) have no influence on postprandial metabolic response in normal weight and overweight/obese patients (analyzed separately); (2) there is no difference in postprandial metabolic response to a particular meal in normal weight and overweight/obese patients. The first hypothesis (1) was verified for two pairs of meals: HC versus NC and HC versus HF for normal weight and overweight/obese subjects. Since both meals were given to the same patients, tests for dependent variables were used: either one-way ANOVA or Wilcoxon signed-rank test (both for paired samples)—depending on fulfilling of the condition of the normality of the variables' distribution. The second hypothesis (2) was verified for each type of meal: HC, NC, and HF in order to check whether there are significant differences in postprandial metabolic response between normal weight and overweight/obese subjects. One-way ANOVA or Wilcoxon rank-sum test (both for unpaired samples)—depending on fulfilling of the condition of the normality of the variables' distribution—and the homogeneity of variances were used to test the stated hypothesis. To address the issue of multiple hypothesis testing, false discovery rate *P* value adjustment method was used. For all calculations, the alpha level was set at 0.05. The areas under the curve (AUCs) were calculated using the trapezoidal method and underwent the same analysis schema as the rest of the features.

## 3. Results

**Figure 2:**
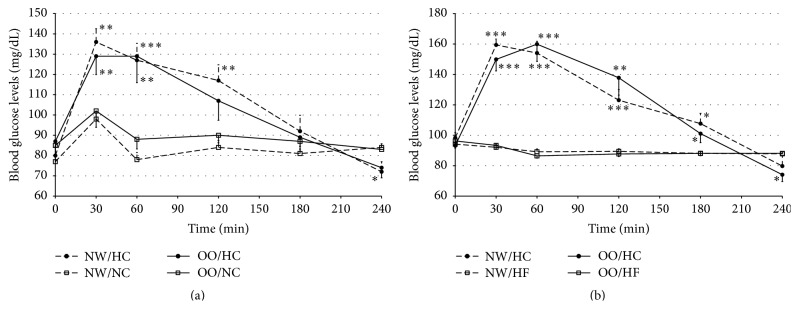
Blood glucose levels (mg/dL) in normal weight (NW) and overweight/obese (OO) men in fasting state (time 0 min) and after (time 30–240 min): (a) high-carbohydrate (HC, black circle) and normal-carbohydrate (NC, white square) meal intake. (b) High-carbohydrate (HC, black circle) and high-fat (HF, white triangle) meal intake. Data are presented as mean value ± SE. Comparison between different meals in NW or OO men: ^*∗*^ *P* < 0.05, ^*∗∗*^ *P* < 0.01, and ^*∗∗∗*^ *P* < 0.001. Comparison between NW and OO men after the same meal intake: ^A^ *P* < 0.05, ^B^ *P* < 0.01, and ^C^ *P* < 0.001.

The blood glucose levels were significantly higher after the HC meal intake than after NC meal at 30 and 60 minutes in OO men and also at 120 minutes in NW individuals (Figure 2(a)). Similarly, the AUCs values were higher after the HC meal intake in comparison to the NC meal, both in NW (25577 ± 1264 versus 20088 ± 402, resp., *P* < 0.01) and in OO men (24994 ± 1687 versus 21345 ± 938, resp., *P* < 0.01).

The blood glucose levels were significantly higher after HC meal in comparison to HF meal as well, both in NW and in OO men (Figure 2(b)). The AUCs for glucose levels were significantly higher after HC meal intake in comparison to the AUCs for glucose levels after HF meal (for NW men 29429 ± 1082 versus 21473 ± 381, resp., *P* < 0.0001, and 29648 ± 1378 versus 21327 ± 331, *P* < 0.0001, for OO men). 240 minutes after HC meal intake the blood glucose levels were significantly lower in comparison to the levels after the remaining meals and glycaemia ≤65 mg/dL was observed in 29% of the participants (6 NW men and 6 OO men; minimal noted glycaemia was 41 mg/dL).

At fasting and postprandial state (Figures 2(a) and 2(b)) blood glucose levels did not differ between NW and OO subjects after any of the meals. The AUCs for blood glucose levels did not differ between NW and OO men after HC meal (*P* > 0.05) and after NC meal (*P* > 0.05) in group I and after HC meal (*P* > 0.05) and HF meal (*P* > 0.05) in group II.

**Figure 3:**
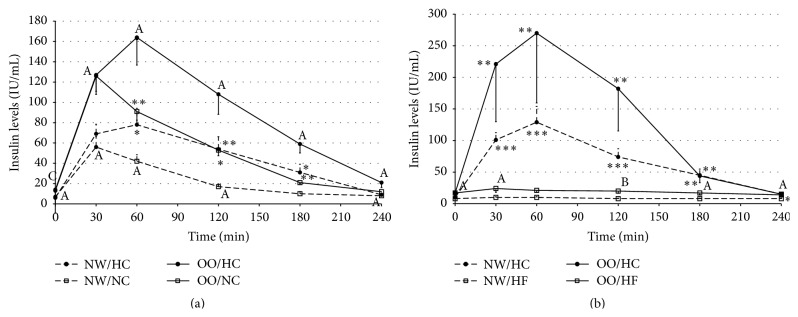
Insulin levels (IU/mL) in normal weight (NW) and overweight/obese (OO) men in fasting state (time 0 min) and after (time 30–240 min): (a) high-carbohydrate (HC, black circle) and normal-carbohydrate (NC, white square) meal intake. (b) High-carbohydrate (HC, black circle) and high-fat (HF, white triangle) meal intake. Data are presented as a mean value ± SE. Comparison between different meals in NW or OO men: ^*∗*^ *P* < 0.05, ^*∗∗*^ *P* < 0.01, and ^*∗∗∗*^ *P* < 0.001. Comparison between NW and OO men after the same meal intake: ^A^ *P* < 0.05, ^B^ *P* < 0.01, and ^C^ *P* < 0.001.

The insulin levels were significantly higher after HC meal in comparison to NC meal, both in NW and in OO men (Figure 3(a)). The insulin levels were significantly higher from 60 to 180 minutes in NW men and in OO men. The values of AUCs for insulin levels were significantly higher after HC meal in comparison to NC meal, both in NW (11047 ± 2162 versus 5694 ± 716, resp., *P* < 0.03) and in OO men (21999 ± 2917 versus 12845 ± 1712, resp., *P* < 0.001).

The insulin levels in NW and OO men were also significantly higher after HC meal intake, in comparison to HF meal (Figure 3(b)). In NW men, the insulin levels were significantly higher from 30 minutes until the end of the tests; however, in OO men insulin levels were significantly higher from 30 to 180 minutes after HC meal intake. The AUCs for insulin levels were higher after HC meal intake than after HF meal, both in NW and in OO men (for NW 16641 ± 2010 versus 2135 ± 149, resp., *P* < 0.0001, and 33075 ± 11070 versus 4533 ± 973, resp., *P* < 0.01, for OO men).

While comparing the insulin levels between NW and OO men, the OO men from group I had significantly higher insulin levels both at fasting state and after HC and NC meals intake (Figure 3(a)). Furthermore, the AUCs for insulin levels after HC and NC meals intake were significantly higher in OO than in NW men (after HC meal 21999 ± 2917 versus 11047 ± 2162, resp., *P* < 0.01; and after NC meal 12845 ± 1712 versus 5694 ± 716, resp., *P* < 0.01). In group II, both at fasting state and after HC meal intake, no differences between insulin levels in NW and OO men were observed (Figure 3(b)), which was most likely due to a high standard error. The insulin levels were significantly higher in OO men before and after HF meal intake (except 60 minutes). Moreover, the AUCs for insulin levels were significantly higher in OO men only after HF meal intake (4533 ± 973 versus 2135 ± 149, resp., *P* < 0.01).

**Figure 4:**
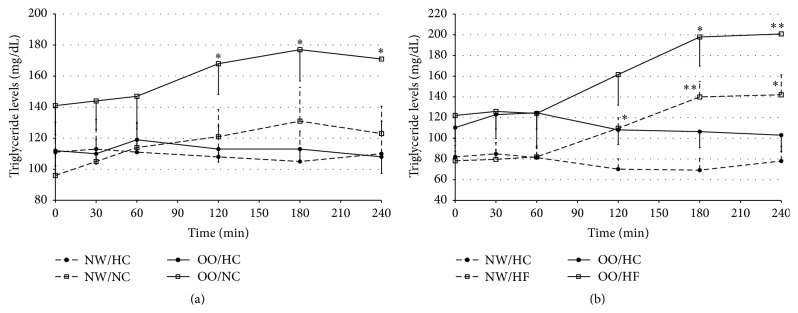
Triglycerides levels (mg/dL) in normal weight (NW) and overweight/obese (OO) men in fasting state (time 0 min) and after (time 30–240 min): (a) high-carbohydrate (HC, black circle) and normal-carbohydrate (NC, white square) meal intake. (b) High-carbohydrate (HC, black circle) and high-fat (HF, white triangle) meal intake. Data are presented as a mean value ± SE. Comparison between different meals in NW or OO men: ^*∗*^ *P* < 0.05, ^*∗∗*^ *P* < 0.01, and ^*∗∗∗*^ *P* < 0.001. Comparison between NW and OO men after the same meal intake: ^A^ *P* < 0.05, ^B^ *P* < 0.01, and ^C^ *P* < 0.001.

In the OO men the TG levels were significantly higher after NC meal at 120, 180, and 240 minutes, in comparison to the levels after HC meal (Figure 4(a)) which was not observed in NW men. The AUCs for TG levels after HC and NC meals in NW men did not significantly differ and amounted to 27592 ± 4785 versus 28382 ± 4344, respectively (*P* > 0.05). In the OO men the AUCs for TG levels were significantly higher after NC meal than after HC meal and amounted to 38888 ± 4640 versus 27057 ± 1879, respectively (*P* < 0.02).

When comparing the TG levels after HC meal with the TG levels after HF meal intake, from 120 to 240 minutes in NW and from 180 to 240 minutes in OO men the TG levels were significantly higher after HF meal intake (Figure 4(b)). The AUCs for TG levels were significantly higher after HF meal in comparison to the AUCs for TG levels after HC meal in NW men (26506 ± 2668 versus 18158 ± 2669, resp., *P* < 0.03). In OO men a tendency for higher values of the AUCs for TG levels after HF meal intake, in comparison to HC meal, was noted (38789 ± 6476 versus 26922 ± 3439, resp., *P* > 0.05).

No differences between NW and OO men (Figures 4(a) and 4(b)) were observed in TG levels nor in the AUCs for TG levels after any of the investigated meals.

**Figure 5:**
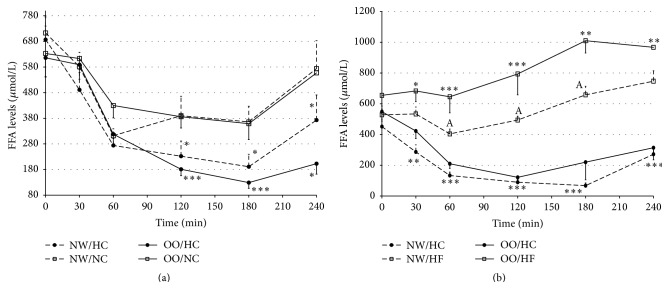
Free fatty acids (FFAs) levels (*μ*mol/L) in normal weight (NW) and overweight/obese (OO) men in fasting state (time 0 min) and after (time 30–240 min): (a) high-carbohydrate (HC, black circle) and normal-carbohydrate (NC, white square) meal intake. (b) High-carbohydrate (HC, black circle) and high-fat (HF, white triangle) meal intake. Data are presented as a mean value ± SE. Comparison between different meals in NW or OO men: ^*∗*^ *P* < 0.05, ^*∗∗*^ *P* < 0.01, and ^*∗∗∗*^ *P* < 0.001. Comparison between NW and OO men after the same meal intake: ^A^ *P* < 0.05, ^B^ *P* < 0.01, and ^C^ *P* < 0.001.

Both, in NW and in OO men, the FFAs levels were significantly higher from 120 to 240 minutes after NC meal intake, in comparison to the levels after HC meal (Figure 5(a)). Moreover, the AUCs for FFAs levels after NC meal were significantly higher than those after HC meal in NW (103435 ± 12984 versus 74272 ± 12380, resp., *P* < 0.03) and OO men (108645 ± 10686 versus 65846 ± 7378, resp., *P* < 0.0001). The differences were more distinct after HF meal intake, when for 30 minutes of testing the FFAs levels were higher in comparison to FFAs levels after HC meal intake, among NW and OO men (Figure 5(b)). The AUCs for FFAs levels were significantly higher after HF meal than after HC meal in NW (133630 ± 6336 versus 38960 ± 5120, resp., *P* < 0.000001) and OO individuals (196624 ± 18147 versus 60300 ± 10017, resp., *P* < 0.0001).

The FFAs levels were similar among NW and OO men, both at fasting and after HC and NC meals intake (Figures 5(a) and 5(b)). However the FFAs levels were significantly higher in OO men in comparison to NW men from 60 to 180 minutes after HF meal intake (Figure 5(b)). The AUCs for FFAs levels did not differ between NW and OO men after HC and NC meals, but significantly higher value of the AUCs for FFAs levels was noted for OO men in comparison to NW men after HF meal intake (196624 ± 18147 versus 133630 ± 6336, resp., *P* < 0.01).

## 4. Discussion

The study confirmed that metabolic response depends on the components of a meal and on the current body energy balance. Moreover, after HF meal intake postprandial hypertriglyceridemia was noted in OO men, even if fasting TG levels were within the normal range and the meal contained predominantly unsaturated fatty acids. It is worth noticing that the investigated study groups comprised healthy young men, and, in spite of this, we observed very early disturbances in postprandial metabolic response, which in long-term can lead to development or progress of metabolic disorders such as obesity, hypertriglyceridemia, metabolic syndrome, and type 2 diabetes.

The highest glucose levels after HC meal, observed in NW men and in OO men, were not surprising and are consistent with the results obtained by other researchers [15, 16]. However, 240 minutes after HC meal intake the glucose levels were significantly lower when compared with the glucose levels after other meals. At the same time, glycaemia ≤65 mg/dL (minimal glycaemia 41 mg/dL) was observed among 29% of the participants, in the same number of NW men and OO men. The mild symptoms typical for hypoglycaemia were observed in most of these people and did not require any medical intervention. On the other hand, both after NC meal and after HF meal intake, glycaemia ≤65 mg/dL was not observed in any of the participants. Taking into consideration the clinical consequences, hypoglycaemia is particularly dangerous for people suffering from diabetes. Hypoglycaemic symptoms are certainly alarming for the patient, and, taking energy homeostasis into consideration, hypoglycaemic episodes can result in weight increase, as sudden and intense hunger constitutes one of the prodromal symptoms. Reactive hypoglycaemia observed after HC meal was most likely related to increased secretion of insulin in response to the significant amount of ingested carbohydrates.

The study showed that glucose levels in general were not different for NW and OO people, both at fasting and after each of the meals. Other researchers also did not observe any significant differences in glucose levels between people with normal weight and those with obesity after high-carbohydrate and high-fat meals intake [14, 17]. However, Zwirska-Korczala et al. [18] observed higher glucose levels among obese people at fasting state, 60 and 120 minutes after a combined meal intake. In our study, increased fasting glucose levels among OO men were observed just once—before NC meal intake.

The insulin levels, both in NW men and in OO men, were significantly higher after HC meal intake than after NC or HF meal. The increased secretion of insulin was undoubtedly a result of higher glucose levels after high-carbohydrate meal, which was also confirmed in studies carried out by other authors [15, 16, 19]. Increased insulin secretion after HC meal intake was most likely the cause of reactive hypoglycaemia observed in some of the participants.

Despite the lack of significant differences in glucose levels, the insulin levels were significantly different both in fasting and in postprandial states. OO men had significantly higher fasting insulin levels after HC, NC, and HF meals intake (in comparison to NW men). Other researchers also observed higher insulin levels, at fasting state and after a meal intake in people with obesity, also with a lack of significant differences in glucose levels [14, 18, 20, 21]. On the other hand, Peake et al. [22] did not observe any differences in postprandial changes of insulin levels in people with normal weight and people with a family history of diabetes and whose BMI categorised them as overweight. In our study, no differences in insulin levels both at fasting state and after HC meal intake between NW and OO men were observed only in group II, but the lack of statistical significance was most likely caused by the high value of standard error, as the average insulin levels in OO people in the first 120 minutes of the test were noticeably higher.

When comparing meals with different composition, we have noticed that in OO men, after NC meal intake, the TG levels were significantly higher in comparison to the levels after HC meal intake. Also among OO men the AUC for TG levels was significantly higher after NC meal, whereas among NW men the AUCs for TG levels after HC and NC meal did not differ significantly. The TG levels were also significantly higher after HF meal in comparison to HC meal, both in NW and in OO men. Other researchers also proved that the high-fat meal intake is related to the increase of TG levels, where the maximum values were obtained 4 hours after ingesting the meal [23]. The higher TG levels in people with normal weight 75 minutes after a high-fat meal intake, in comparison to a high-carbohydrate meal, were observed also by Raben et al. [15]. We did not observe any differences in TG levels between NW and OO people. It is worth noticing that after NC and HF meals intake a postprandial hypertriglyceridemia was observed in OO people. The study conducted by Peake et al. [22] also did not present any differences in TG levels after a high-fat meal between people with normal weight and people with a family history of diabetes and whose BMI categorised them as overweight. In our study, after HF meal rich in unsaturated fatty acids, which are recommended due to their beneficial effect on the risk of coronary heart disease [6, 24], we have noted postprandial hypertriglyceridemia in OO men, even though the average fasting TG levels remained in a normal range. As the studies show, postprandial dyslipidaemia is related to the intensification of inflammatory disease processes, vascular endothelial dysfunction, impaired fibrinolysis, platelet instability, risk of coronary artery disease, and cardiovascular incidents such as myocardial infarction, ischemic stroke, and death [3, 25, 26]. Postprandial hypertriglyceridemia could be observed due to a high total fat content in the meal but also due to a high content of MUFA, since in some studies there were observed benefits of MUFA on cardiovascular risk factors [27]. However, other studies showed that monounsaturated fatty acids appeared not to have cardioprotective effect and, what is more, they showed that MUFA intake may be associated with increased risk of fatal coronary heart disease [28, 29].

Also the high FFAs levels are responsible for elevated endothelial activation markers, vascular endothelial cell inflammation, and increased prothrombotic activity markers, which can cause vascular and atherosclerotic anomalies resulting in circulatory system diseases [30]. As a part of this study, the FFAs levels were evaluated and it was observed that in NW men, as well as in OO men, FFAs levels were significantly higher after NC and HF meals intake, in comparison to HC meal. FFAs levels were decreasing in relation to the fasting values after HC meals, and after NC meals they were decreasing to a lesser degree. Raben et al. [15] also observed lower FFAs levels after a high-carbohydrate meal intake, in comparison to other meals in people with normal weight. FFAs, if not metabolized for energy, can be deposited as triglycerides in adipose tissue which results in obesity [31]; they can also be accumulated ectopically in muscle tissue, heart muscle, or liver, as a possible defence mechanism against lipotoxicity, which can cause cell dysfunction and death [32]. The ability to store lipids by cells other than adipocytes may be limited and ectopic accumulation of lipids can result in insulin resistance and type 2 diabetes mellitus [33]. The correlation between increased FFAs availability and impaired glucose metabolism is well established and it seems that several mechanisms can be involved in this process. The excess FFAs availability may disrupt glucose metabolism through the competition between substrate utilization, through lipid induced changes in the phosphorylation and function of proteins in the pathway of insulin signalling for GLUT4 translocation, but also other mechanisms have been proposed and discussed [34].

The conducted analyses did not show any significant differences in fasting FFAs levels between NW and OO people, and the AUCs for FFAs levels after HC and NC meals were the same for NW and OO people as well. However, 60 minutes after HF meal intake higher FFAs levels were observed among OO people in comparison to NW men and remained significantly higher until 180 minutes of the test. Also the AUC of FFAs levels in postprandial state after a high-fat meal intake was significantly higher in OO than in NW men. Imbeault et al. [14] also observed an increase in FFAs levels after a high-fat meal and significantly higher FFAs levels in obese people. Other researchers [22] did not find any differences in FFAs levels after a high-fat meal intake between people with normal weight and overweight ones. The results are not unambiguous and require further studies on larger study groups.

The differences in postprandial metabolic effects, especially in lipids response, probably can be explained by the lower numbers of lipoprotein particles of hepatic origin that compete with chylomicrons for lipolysis [35], which result in improved postprandial TG metabolism in NW individuals. Another mechanism may be related to insulin sensitivity, which stimulates lipoprotein lipase activity and TG clearance, and, as it was shown, by the weight loss (which improves insulin sensitivity) the fat-induced postprandial TG clearance was improved as well [36]. Some previous studies have shown that insulin resistance is associated with increased fatty acids levels released by adipose tissue and elevated VLDL-TG secretion in the hepatocytes and lower adipose tissue lipoprotein lipase activity [37, 38]. Therefore, the higher postprandial insulin levels noted in OO men might be related to the lower postprandial insulin sensitivity, and it can be an explanation of observed higher TG response.

Moreover, the postprandial response can be dependent also on the other, for example, genetic factors, which we have observed in one of our previous studies [39].

In summary, the conducted study confirmed that postprandial metabolic response depends on the components of a meal and moreover it is different among NW and OO people.

The highest blood glucose levels were observed after HC meal intake; however the levels were the same for NW and OO people. Insulin levels were also the highest after HC meal intake, and, despite the lack of significant differences in blood glucose levels, higher insulin levels were observed in OO people, in comparison to the NW group. Triglyceride and FFAs levels were higher after NC and HF meals intake. In OO people, postprandial hypertriglyceridemia was observed after HF meal intake, even if it contained mostly unsaturated fatty acids. Further investigations on the effect of MUFA on the CHD risk are undoubtedly warranted. Nevertheless, our findings showed that metabolic response in OO people to high-fat meals, even if rich in unsaturated fatty acids, caused postprandial hypertriglyceridemia and should be avoided in diet. Even if the study sample size was quite small and our results need to be confirmed, for example, by increasing the sizes of study groups, the obtained results have crucial significance in a period of obesity plague and of the omnipresent trends of diets with modified amount of macronutrients, including high-fat diets.

## Figures and Tables

**Table 1:** The characteristic of study groups.

Group I	NW (*n* = 11)	OO (*n* = 12)	*P*
Age, years	33 ± 2	40 ± 2	<0.02
BMI, kg/m^2^	23.8 ± 0.5	31.4 ± 1.5	<0.001
Body fat content, %	17.9 ± 1.0	28.6 ± 1.7	<0.0001

Group II	NW (*n* = 12)	OO (*n* = 11)	*P*

Age, years	33 ± 3	36 ± 3	>0.05
BMI, kg/m^2^	23.9 ± 0.2	33.7 ± 2.2	<0.00001
Body fat content, %	18.6 ± 1.5	31.9 ± 2.7	<0.001

Data are presented as a mean value ± SE.
